# High-Frequency Stimulation of Nucleus Accumbens Changes in Dopaminergic Reward Circuit

**DOI:** 10.1371/journal.pone.0079318

**Published:** 2013-11-14

**Authors:** Na Yan, Ning Chen, Honghua Zhu, Jianguo Zhang, Moira Sim, Yu Ma, Wei Wang

**Affiliations:** 1 Beijing Municipal Key Laboratory of Clinical Epidemiology, School of Public Health, Capital Medical University, Beijing, China; 2 Department of Neurosurgery, Beijing Sanbo Brain Hospital, Capital Medical University, Beijing, China; 3 Department of Gastroenterology, The Sixth People Hospital of Jinan, Jinan, China; 4 Department of Neurosurgery, Beijing Tiantan Hospital, Capital Medical University, Beijing, China; 5 Systems and Intervention Research Centre for Health, School of Medical Sciences, Edith Cowan University, Perth, Western Australia, Australia; 6 Yuquan Hospital, Tsinghua University, Beijing, China; Peking University, China

## Abstract

Deep brain stimulation (DBS) of the nucleus accumbens (NAc) is a potential remedial therapy for drug craving and relapse, but the mechanism is poorly understood. We investigated changes in neurotransmitter levels during high frequency stimulation (HFS) of the unilateral NAc on morphine-induced rats. Sixty adult Wistar rats were randomized into five groups: the control group (administration of saline), the morphine-only group (systematic administration of morphine without electrode implantation), the morphine-sham-stimulation group (systematic administration of morphine with electrode implantation but not given stimulation), the morphine-stimulation group (systematic administration of morphine with electrode implantation and stimulation) and the saline-stimulation group (administration of saline with electrode implantation and stimulation). The stimulation electrode was stereotaxically implanted into the core of unilateral NAc and microdialysis probes were unilaterally lowered into the ipsilateral ventral tegmental area (VTA), NAc, and ventral pallidum (VP). Samples from microdialysis probes in the ipsilateral VTA, NAc, and VP were analyzed for glutamate (Glu) and γ-aminobutyric acid (GABA) by high-performance liquid chromatography (HPLC). The levels of Glu were increased in the ipsilateral NAc and VP of morphine-only group versus control group, whereas Glu levels were not significantly changed in the ipsilateral VTA. Furthermore, the levels of GABA decreased significantly in the ipsilateral NAc, VP, and VTA of morphine-only group when compared with control group. The profiles of increased Glu and reduced GABA in morphine-induced rats suggest that the presence of increased excitatory neurotransmission in these brain regions. The concentrations of the Glu significantly decreased while the levels of GABA increased in ipsilateral VTA, NAc, and VP in the morphine-stimulation group compared with the morphine-only group. No significant changes were seen in the morphine-sham stimulation group compared with the morphine-only group. These findings indicated that unilateral NAc stimulation inhibits the morphine-induced rats associated hyperactivation of excitatory neurotransmission in the mesocorticolimbic reward circuit.

## Introduction

Opiate drugs such as morphine are commonly used to relieve pain. However, this class of psychoactive agents can also elicit intense euphoric effects followed by feelings of well-being in the user, particularly when taken in high doses, and this can lead to drug abuse and ultimately addiction [1]. Although drug withdrawal syndrome can be alleviated by means of pharmacotherapy and psychotherapy, the effects of such approaches on drug craving, which induces relapse, are usually minimal. The mesocorticolimbic dopamine reward circuit, which consists of the ventral tegmental area (VTA), nucleus accumbens (NAc), and ventral pallidum (VP), plays a critical role in drug addiction [2]–[4]. NAc has a central role in the pathogenesis of drug dependence and is an important element in the mesocorticolimbic reward circuit [2], [5]–[6]. NAc is centrally involved in establishing the reward and salience of drugs of abuse [7], and is therefore an attractive target for treatment. In a clinical study bilateral NAc lesions induced by stereotactic surgery alleviated opiate drug psychological dependence [8]. However, in view of the invasiveness, irreversibility, severity of adverse effects, and ethical controversy, ablation surgery cannot be accepted as a routine treatment. Deep brain stimulation (DBS) has the advantages of being reversible, adjustable, and minimally invasive, and has become a potential treatment in neurobiologically-based interventions for addiction. Xu *et al.* reported that bilateral NAc-DBS in an opiate drug-dependent patient had excellent effects and relapse decreased significantly without significant complications [9].

Nonetheless, the mechanism underlying NAc-DBS treatment of addiction remains unclear. It was reported that addiction may be related to an imbalance between the excitatory glutamate (Glu) and inhibitory γ-aminobutyric acid (GABA) neurotransmitters [10]–[12]. Some studies suggested that glutamate release in the NAc is necessary for heroin and cocaine craving [13], [14]. However, other studies demonstrated that changes in GABA levels might be linked to the motivational aspects of drug addiction [15]. High frequency stimulation (HFS) of deep brain regions such as globus pallidus internus (Gpi) or subthalamic nucleus (STN) can reverse *Parkinson’*s symptoms through the activation of GABAergic neurotransmission and increased inhibitory neurotransmitter GABA release, and consequently attenuate dopamine release [16]–[18]. However, it is not known whether NAc stimulation might operate via similar pathways.

The conditioned place preference (CPP) is one of the most established experimental protocols which used for measuring drug reward in laboratory [19]. Based on Pavlovian conditioning principles, CPP reflects a preference for a context due to the contiguous association between the context and a drug-associated stimulus [20]. All drugs of abuse could increase the conditioning time in this protocol [21]. Therefore this is a simple and effective method to assess the rewarding properties of drugs [19], [21].

The present study aimed to evaluate the effects of NAc-DBS on changes of Glu and GABA levels in NAc, VTA, and VP of morphine-induced addiction rats produced by systemic morphine administration. Intracerebral microdialysis was used to monitor the extracellular concentrations of Glu and GABA in the ipsilateral NAc, VTA, and VP.

## Materials and Methods

### Animals

Sixty adult male Wistar rats weighing 280–320 g were provided by the Experimental Animal Center of Military Medical Sciences, Beijing, China. The animals were individually housed in cages (constant temperature, 22–25°C; humidity, 60–70%; illumination, 12/12 h cycles) with free access to food and water. All experiments were performed during the light phase of the cycle. These were randomized into five groups, all of which had intraperitoneal injections: (1) the control group (n = 12), which underwent administration of saline; (2) the morphine-only group (n = 12), which underwent administration of morphine without electrode implantation; (3) the morphine-sham-stimulation group (n = 12), which underwent administration of morphine with electrode implantation but not given stimulation; (4) the morphine-stimulation group (n = 12), which underwent administration of morphine with electrode implantation and HFS stimulation, (5) the saline-stimulation-group (n = 12),which underwent administration of saline with electrode implantation and stimulation. The experimental procedure is shown in Fig. S1. Care and handling of animals were conducted in compliance with the Chinese Animal Welfare Act and the study was approved by the Ethical Committee of Capital Medical University, Beijing.

### Drugs

Rats in the morphine-only, the morphine-sham-stimulation and the morphine-stimulation groups received intraperitoneal injections of morphine hydrochloride (lot 080403, Shengyang Pharmaceutical Factory, China) at increasing doses (10, 20, 30, 40, 50, 60 mg/kg, then fixed at 60 mg in physiological saline once per day. The injection was applied 10 minutes prior to the start of the conditioning experiment every day until morphine-seeking behavior occurred, i.e. rats spent more time in the drug-paired compartment. Rats in the control group and saline-stimulation group received the same doses of physiological saline.

### Surgical Procedures

**Figure 1:**
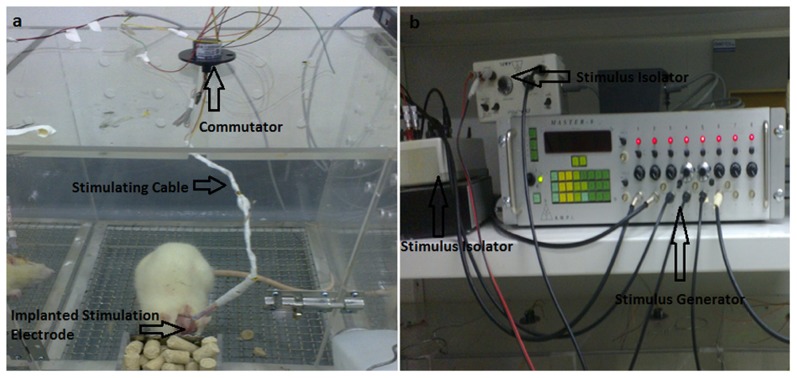
Phograph of an individual rat undergoing NAc-DBS. An Signal Generator connected to a Constant Current Bipolar Stimulus Isolator that is connected to the concentric bipolar electrode implanted into the rat NAc and secured using dental cement and bone screws. The stimulator cable is routed through a commutator to allow the rat to move freely during the stimulation phase.

The animals was anesthetized with chloral hydrate (350 mg/kg, i.p.) and placed in a stereotaxic apparatus. A microdialysis guide cannula with a stylet (Bioanalytical Systems, USA) was implanted. The concentric bipolar stimulation electrodes (outer diameter 200 µm, inner diameter 50 µm, CBBPF50, FHC, USA) were glued to the guide cannula with a specially made device, and the electrode tips were 2 mm below the end of the guide cannula. Four cannulas were implanted in each rat: one unilaterally in the core of the NAc (for stimulation microelectrode, AP = +1.7 mm, L = +2.5 mm at an angle of 6°, V = –5.5 mm); one within the ipsilateral core of NAc (for microdialysis, AP = +1.8 mm, L = +1.2 mm, V = –6.5 mm); one within the ipsilateral VTA (AP = –5.2 mm, L = +0.6 mm, V = 8.4 mm); and one within the ipsilateral VP (AP = –0.5 mm, L = +2.8 mm, V = –6.2 mm) according to the atlas of Paxinos and Watson [22]. The cannulas were cemented in place using dental cement to affix them to three stainless steel screws fastened to the skull. Following surgery, animals were housed individually in Plexiglas cages immediately. After a recovery of seven days, continuous unilateral NAc stimulation was then performed using a stimulator system (Master-8 and ISO-flex, Israel) while the electrical stimulation was turned off during both the saline- and morphine-pairings (Fig. 1). Parameters were: intensity 2 V, pulse width 60 µs, frequency 130 Hz. Only the morphine-stimulation and saline-stimulation groups received stimulation; the morphine-sham-stimulation group was manipulated in the same way but without electrical stimulation.

### Conditioned Place Preference (CPP)

CPP apparatus (Tracksys, Nottingham, UK) were three-chambered shuttle boxes comprising a small central compartment (10×10 cm) where rats were placed at the start of a test session, and two larger compartments (40×40 cm), one with horizontal black and white stripes and one with vertical black and white stripes. Floors were made of stainless steel sheeting with punched-out shapes(circles, 12 mm hole and squares, 10 mm hole) resulting in distinct textures (Novametals, Manchester, UK). Removable partitions allowed the boxes to be used either to restrict the rats to a particular compartment for conditioning or to allow the rats to be ‘free-to-explore’ during a test session. Experiments were performed between 8∶00 am and 6∶00 pm under dim white light (light intensity approximately 15l ux). During all test sessions, the time each rat spent in each compartment was recorded using EthoVision XT (Noldus Information Technology, Utrecht, the Netherlands) tracking software.

### Morphine Conditioning and Behavioral Testing

Conditioned place preference consisted of a 14-day schedule with three phases: pre-conditioning, conditioning and post-conditioning. The compartment occupied for the shorter time was designated as the drug-paired compartment. Previous work in our laboratory indicated that naïve rats tend to display a preference for the black one. Therefore the white chamber was designated as the drug-paired compartment that is the site where morphine would be administered.

Pre-conditioning phase: during this phase, each animal from all groups was placed in compartment with the guillotine door removed to allow access apparatus for 900 sec each day for three consecutive days. On day 3, the time rats spent in each compartment was recorded.

Conditioning phase: Conditioning sessions were conducted from day 4 to day 13 with each group receiving 6 hourly alternating injections of either saline or morphine. Each conditioning session was 30 minutes in duration, once daily. From 8∶00 to 12∶00 on the first training day, rats in each group were injected with saline and immediately confined to the black compartment (not drug-paired side) of the shuttle box; from 14∶00 to 18∶00 the rats were confined to the white compartment (drug-paired side) after the saline was administered to the control and saline-stimulation group and morphine to the morphine-only group, morphine-stimulation group and morphine-sham-stimulation group. On the second day, the procedure was performed in reverse order, that is, from 8∶00 to 12:00 the rats were confined to the white one after the saline was administered to the control and saline-stimulation group and morphine to the morphine-only group, morphine-stimulation group and morphine-sham-stimulation group; from 14∶00 to 18∶00 the rats in each group were injected with saline and confined to the black one.

Post-conditioning or testing phase. This phase was carried out 1 day after the last training in a morphine-free state. On the test day, the removable wall was raised and the rat could access the entire apparatus for 900 seconds. Conditioned place preference was indicated by a preference score, defined as the time spent in the drug-paired side minus the time spent in the saline-paired chamber.

### Microdialysis

One day after the post-conditioning phase, each rat was lightly anesthetized with pentobarbital and the three guide stylets were replaced with the microdialysis probes leaving the stimulation electrodes in place (CMA12, Sweden). The probes were perfused with artificial cerebrospinal fluid (NaCl, 145 mM; KCl, 3.8 mM; MgCl_2_, 1.2 mM; CaCl_2_, 1.2 mM; pH 7.4) at a flow rate of 2 ml/min through a microsyringe pump (CMA400, Sweden). The dialysis tubing was connected to a liquid swivel for collection from the lightly anesthetized animals. A 2-h stabilization period was allowed before the collection of dialysates for analysis, and dialysate fractions were then collected at 30 min intervals. The three fractions were collected during stimulation. Dialysates were automatically collected with a refrigerated autosampler and stored at –80°C until analysis.

#### Determination of neurotransmitter levels

**Figure 2:**
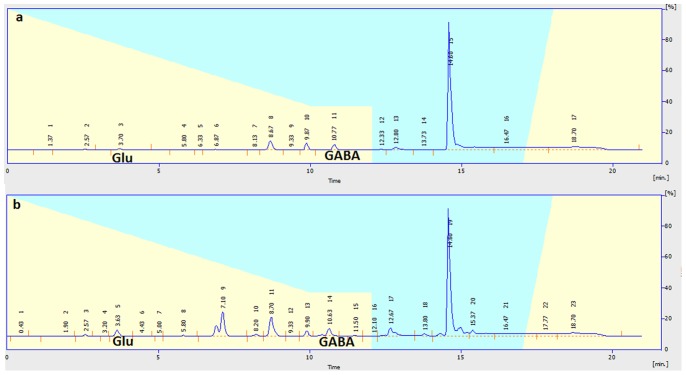
The chromatogram of Glu and GABA. a: standard substance; b: sample.

Neurotransmitter (Glu and GABA) levels in the dialysate samples were determined by high-performance liquid chromatography (HPLC) with fluorometric detection. Briefly, samples or standards were derivatized with O-phthaldialdehyde; 20 µl of the resulting mixture was automatically loaded onto a Novapark C_18_ reverse-phase column (150×3.9 µM, 4 µm particle size; Waters) using a refrigerated autoinjector. The mobile phase consisted of NaH_2_PO_4_ (0.05 M, pH 6.8) with 20% methanol at a flow rate of 1 ml/min delivered by a Waters pump. Glutamate and GABA were identified through their migration time and spike profiles. Peaks values were normalized to an internal standard curve for fluorescein and quantified by comparison with an external standard curve (analytical software: EMPOWER) (Fig. 2). The values were corrected for in vitro probe recovery, which was approximately 10%.

### Histological Examination

After completion of all experiments, the rats were anesthetized with urethane (1 g/kg) and perfused transcardiac with 0.1 M phosphate-buffered saline followed by 4% paraformaldehyde. Brains were removed and coronal sections (30 µm) through the target were prepared and processed by hematoxylin and eosin (HE) and cresol violet staining to confirm the location of the stimulating electrode. Only rats with electrode and probe placements in the areas of interest were included in subsequent data analysis.

### Statistical Analysis

Results were expressed as means ±SEM. Repeated measures were used to determine whether the mean values of Glu and GABA at the indicated time during the unilateral NAc-DBS differed significantly from basal values. Statistical analysis employed one-way analysis of variance (ANOVA) followed by Tukey test for multiple *post hoc* comparisons using SPSS 13.0 analytical software for Windows (SPSS Inc., Chicago, IL,USA). A *P* value of less than 0.05 was considered statistically significant.

## Results

### Effect of High-frequency DBS on Morphine-induced CPP

**Figure 3:**
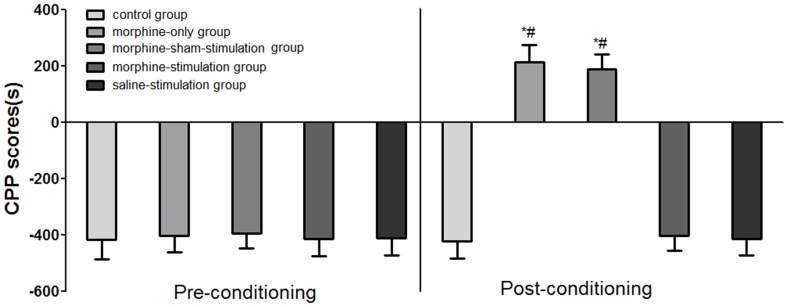
Effects of exposure to high frequency stimulation on morphine-induced conditioned place preference (CPP). Data represent means ± SEM for time spent on the drug-paired minus time spent on the saline-paired side; *P<0.05, compared with control group; #P<0.05, compared with the morphine-stimulation group.

At baseline (pre-conditioning), mice in each group spent a longer time on the black sides of the chamber than the white sides of the chamber (F _(4, 56)_ = 0.2134; P>0.05; Fig. 3), reflecting the usual preference for the black compartment shown by laboratory naïve rats. So we chose their non-preferred chamber (white sides) as the drug-paired room and the preferred chamber (black sides) as the saline paired room. In the period of morphine administration, rats in the morphine-only group and the morphine-sham-stimulation group spent increasing time in the white compartment. However, the time that rats in the morphine-stimulation group or saline-stimulation group spent in the white compartment (drug-paired) did not increase. Time spent in the white sides of the rats in the control group (administration of saline only) also did not increase. Effects of exposure to stimulation on morphine-induced CPP during the conditioning procedure are shown in Fig. 3. Results showed that administration of morphine without electrical stimulation was associated with an increase in time spent in the drug-paired compartment in the morphine-sham-stimulation group (187.04±53.45 seconds) and morphine-only group (203.92±58.53 seconds), respectively, compared with the control group (−422.94±59.6 seconds) (F_ (4, 56)_ = 396.1, P<0.05). However exposure to 130 Hz high frequency stimulation was not associated with a significant increase in the time spent in the drug-paired sides in the morphine-stimulation group (−403.28±53.25 seconds) and the saline-stimulation group (−415.04±57.2 seconds) compared with the control group (−422.94±59.6 seconds) (F _(4, 56)_ = 396.1, P<0.05). Our data demonstrated that 130-Hz electrical stimulation of NAc significantly blocks morphine-induced CPP.

### Neurotransmitter Levels in VTA, NAc, and VP

Significant increases in the levels of Glu in NAc and VP of the morphine-only groups and morphine-sham-stimulation group were observed when compared to the control (*P*<0.05). No significant differences were observed in the levels of Glu in the VTA of the morphine-only, the morphine-sham stimulation group and the control groups (*P*>0.05). In the morphine-sham-stimulation group, no significant differences in the extracellular concentrations of Glu in NAc, VP and VTA were found compared with the morphine-only group (*P*>0.05). However, we observed that significant decline in the levels of Glu in NAc, VP and VTA in the morphine-stimulation group when compared with the morphine-only, morphine-sham-stimulation, saline-stimulation and control groups (*P*<0.05) (Table 1).

**Table 1:** Levels of Glu and GABA in dialysates in the VTA, NAc and VP collected in each group (Mean ± SEM, nM/ml).

group	n	NAc		VP		VTA	
		Glu	GABA	Glu	GABA	Glu	GABA
control group	11	1.76±0.37#	1.89±0.45#	1.56±0.35#	2.45±0.33#	1.78±0.31#	1.42±0.32#
Morphine-only group	11	3.54±0.41* #	1.05±0.32* #	2.92±0.48* #	1.57±0.38* #	1.87±0.29#	1.03±0.27* #
morphine-sham-stimulation group	11	3.47±0.39* #	1.08±0.27* #	2.86±0.34* #	1.69±0.41* #	1.75±0.36#	1.05±0.37* #
morphine-stimulation group	12	1.17±0.29*	3.23±0.35*	1.07±0.32*	3.07±0.26*	1.23±0.27*	2.49±0.34*
saline-stimulation group	11	1.63±0.233#	1.98±0.32#	1.49±0.31#	2.56±0.26#	1.64±0.34#	1.53±0.28#
F		117.3	75.87	61.64	40.84	7.428	40.56
P		<0.0001	<0.0001	<0.0001	<0.0001	<0.0001	<0.0001

Statistical analysis was performed by one-way ANOVA followed by post hoc analysis.

* *P*<0.05, compared with control group;

# *P*<0.05, compared with morphine-stimulation group.

In the case of GABA, we observed that significant decline in NAc, VTA and VP, respectively, of the morphine group and morphine-sham-stimulation group, respectively, compared to the control group (*P*<0.05). In the morphine-sham-stimulation group, we did not find statistical significance in the extracellular concentrations of GABA in NAc, VP and VTA, respectively, compared with the morphine group (*P*>0.05). However, we observed that significant increase in the levels of GABA in NAc, VP and VTA, respectively in the stimulation group when compared with the morphine, morphine-sham stimulation, saline-stimulation and control groups (*P*<0.05) (Table 1).

### Histology

**Figure 4:**
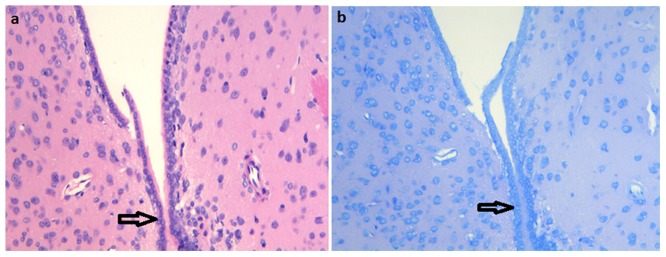
Histological examination in the morphine-stimulation group. a: HE staining(x200); b: Nissl staining(x200).

In total, sixty animals were used. At the end of all behavioral experiments, the location of the cannula was examined histologically. Histological analysis demonstrated that the location of the electrode tips showed an identical pattern of distribution within or close to NAc, VP, and VTA in 56 animals. Another 4 animals were not included in the statistical analyses due to the mislocation or misplacement of cannulae. In addition, coronal brain sections indicated no significant cell necrosis or neuronal loss in the NAc surrounding the lead contact in the the morphine-stimulation group (Fig. 4).

## Discussion

To address the mechanism underlying the use of DBS as a potential therapy for morphine induced rats, we investigated the DBS effects of NAc on GABAergic and glutamatergic activity within the mesocorticolimbic system. Successful induction of a state of morphine-conditioned preference in rats treated with systematic administration of morphine was confirmed in a CPP test. Using microdialysis probes implanted into the ipsilateral NAc, VTA, and VP, we then measured levels of the neurotransmitters Glu and GABA.

The study showed that morphine-conditioned status was accompanied by a characteristic pattern of changes in the brain tissue concentrations of these neurotransmitters. The levels of Glu increased in the NAc and VP of morphine-only group compared to that of the control group, although the level of Glu in the VTA did not change significantly. However, levels of GABA decreased significantly in all three regions (NAc, VP, and VTA) of morphine-only group versus control group. These results demonstrated that rats with morphine-conditioned status displayed neurotransmitter changes associated with heightened activation of excitatory pathways in mesocorticolimbic structures.

The study also demonstrated that high-frequency electrical stimulation of the unilateral NAc could substantially reverse these changes. Compared to the morphine group, the levels of Glu decreased significantly in NAc, VTA, and VP, whereas GABA levels increased significantly in all three regions in the morphine-stimulation group. The result suggested that these changes could underlie the utility of DBS in the treatment of morphine-induced rats.

Previous studies indicated that the effects of NAc stimulation on morphine-induced addiction were frequency-dependent. High-frequency stimulation (HFS; 130 Hz) of the NAc was more effective than low-frequency stimulation (LFS; 20 Hz) in treating morphine induced rats [23]. In the current study we selected 130 Hz for NAc stimulation. The precise region of the NAc for stimulation has not been rigorously established. NAc comprises two principal subregions, the central core and peripheral shell. In general, the former is associated with extrapyramidal motor control, and the latter with the limbic system. Within NAc, information is transmitted from shell to core. Together with the ventral striatum, NAc, particularly the shell region, receives a strong dopaminergic input from VTA. NAc thus attains a central position between limbic and mesolimbic dopaminergic structures. It was reported that the core of NAc is necessary for drug-seeking behavior [24]–[25]. We therefore implanted stimulation electrodes into the core region of the NAc. Because that unilateral HFS of NAc is effective in morphine-induced addiction rats [26], we performed stimulation on one side and recovered microdialysis samples from the ipsilateral tissues.

### Basal Neurotransmitter Levels in Morphine-induced Rats

An imbalance between GABA and Glu neurotransmission was hypothesized to produce neuronal hyperexcitability in addiction, and it was previously suggested that increased levels of extracellular Glu and reduced levels of extracellular GABA are key factors in drug addiction [27], [28]. Our results were consistent with the above reports. Glu levels were significantly increased in the NAc and VP of morphine-induced rats, although no significant changes were seen in VTA. By contrast, significant decreases in GABA levels were recorded in all three regions. Together, these data supported that the contention that morphine-induced addiction status in these animals was accompanied by a shift in the excitatory/inhibitory balance and hyperactivation of mesocorticolimbic neuronal networks.

Glu is the most prominent excitatory neurotransmitter in the brain. Following release into the synaptic cleft, Glu binds to and activates NMDA- and/or AMPA-type glutamate receptors, leading to onward propagation of action potentials. It was thought that excess glutamatergic neuronal activity may underlie drug addiction [29]–[30], and previous work demonstrated that opioid dependence, withdrawal, craving and relapse are associated with aberrant glutamatergic neurotransmission [31]–[32]. Specifically, prelimbic glutamatergic projections to the NAc and VP were suggested to underlie drug-seeking behavior for many classes of addictive drugs [25], [33]–[34]. Extracellular Glu levels were reported significant elevated in the NAc of addiction rats [35], [36]. An increase in Glu levels was also reported in rats self-administering heroin or cocaine [37], [38]. However, there was a report that extracellular Glu levels did not significantly change in the VTA of rats repeatedly treated with morphine [39]. In this study we confirmed that Glu was increased markedly in both NAc and VP of the morphine-addiction rats, but there were no significant changes in VTA.

Our study also showed that morphine-addiction status in rats was accompanied by a significant decrease of extracellular GABA levels in NAc, VP, and VTA. GABA is the most important inhibitory neurotransmitter in the brain. According to the prevailing hypothesis that dopamine released in the NAc inhibits GABAergic medium spiny neurons projecting to VP, this process is thought to represent a final common path for drug reward [40]. Depression of the output of medium spiny neurons, in other words suppression of GABA release, appears to be a common feature of several drugs of abuse and could be important for their reinforcing properties [41]. In accord with the hypothesis that morphine behavioral reinforcement is linked to its suppression of GABA release, we reported that morphine-addiction status was accompanied by significantly reduced levels of GABA in NAc, VP, and VTA. Others studies demonstrated that amphetamine, cocaine, and heroin suppressed extracellular GABA levels in VP [37], [42]–[43]. Klitenick *et al.* demonstrated that GABA levels fell after morphine administration directly into VTA via a dialysis probe [44]. It was also suggested that activation of μ-opioid receptors located on GABAergic interneurons is the principal mechanism mediating the actions of morphine and other μ-opioids on dopaminergic transmission in the VTA [45]. Morphine binding to receptors on VTA GABA interneurons is thought to inhibit GABA release and decrease tonic inhibition of VTA dopaminergic neurons, thereby leading to increased firing and increased dopamine release in NAc, and in turn decreasing GABA release in the NAc [46]. Our results were in agreement with these views, as well as with earlier findings which suggested a role for striatopallidal mechanisms in mediating drug reward and a role for pallidal GABAergic transmission in the control of morphine addiction [4].

It was reported that inhibiting extracellular Glu release or NMDA receptor expression in the NAc of morphine-treated rats, or increasing GABA concentrations in VTA or VP, could reduce opioid dependence [47]. Together, these findings argue that a therapy of decreasing neuronal excitability in the mesocorticolimbic dopamine reward circuit may be beneficial in controlling drug addiction.

### Effects of NAc Stimulation on Extracellular Neurotransmitter Levels

NAc stimulation as a means to control drug addiction has shown promising results in both animal models and clinical trials. The reported low incidence of adverse events with this procedure is also encouraging. However, the mechanism by which NAc electrical stimulation might provide a treatment for drug addiction is not fully understood. Given that drug addiction in several models has been linked to increased mesolimbic activation, it is notable that, in morphine-CPP rats, unilateral NAc stimulation led to significant reductions in extracellular ipsilateral levels of Glu in NAc, VP, and VTA. Concomitantly, stimulation led to a marked rise in extracellular GABA concentrations in all three regions in our study. An obvious interpretation is that NAc stimulation dampens mesocorticolimbic hyperactivation in morphine-conditioned rats. Indeed, it seems likely that effects on neurotransmitter release may be one mechanism underlying the addiction-aborting effects of NAc stimulation.

However, it has been speculated that electrical stimulation of the NAc might exert its effects by inducing lesions at the point of implantation that are provoked by the implantation of the electrode. However, we controlled for this in our study by employing an implanted electrode without electrical stimulation. The presence of an electrode implanted into NAc failed to change extracellular levels of either neurotransmitter. In addition, coronal brain sections indicated no significant cell necrosis or neuronal loss in the NAc around the lead contact. These findings, consistent with experiment [48], indicated that the placement of the electrode or prolonged low-voltage stimulation caused no permanent marked tissue damage or neuronal loss in the NAc.

Earlier studies showed that HFS can suppress somatic neuronal activity [49], [50] and block axonal conduction. These inhibitory effects were associated with reduced neuronal excitability, increased inhibitory neurotransmission, and depression of excitatory neurotransmission [51]. HFS-induced prolonged release of GABA could result from increased recruitment close to the target of local GABAergic connections including GABAergic fibers from other brain regions [52], [53]. Alternatively, inhibition of presynaptic GABA transporters could lead to reduced reuptake of GABA and increased extracellular GABA levels [54].

Overall, HFS of NAc is thought to inhibit the activity of NAc neurons, and this could underlie the efficacy of NAc stimulation in alleviating drug addiction [23]. Nevertheless, *in vitro* stimulation affects only local neurons, and not more distal neurons [55] and it is possible that local HFS only reduces the activity of a subfraction of NAc neurons. We suspect that, possibly in addition to local inhibition, the efficacy of HFS may depend on projections that leave the area of stimulation and extend to other CNS structures [23]. The neuronal population in NAc is principally composed of GABAergic projection neurons [56], but accumbal extracellular GABA levels are predominantly derived from collaterals of the ventral striatal GABAergic neurons and/or GABAergic afferents from the VTA which project to VP and could represent a final common pathway for drug and other rewards [40], [57], [58]. Therefore, HFS-mediated suppression could operate by increasing GABA release from NAc to VP, and inhibit VP neuronal activation. VTA also receives GABAergic efferents from NAc [59], and NAc HFS could increase GABA release within VTA, therefore decrease dopamine release. In addition, VTA, NAc, and VP receive glutamatergic projections from the prefrontal cortex [33], [34]. We speculate that HFS of NAc may lead to decreased Glu levels in ipsilateral VTA, NAc, and VP by inhibiting the neuronal soma of the associated prefrontal cortex. By this mechanism unilateral HFS stimulation of the NAc could reduce ipsilateral Glu output, leading to disruption of the addiction excitatory network.

Microdialysis has been widely used to sample GABA and Glu from brain. It has, however, been pointed out that there may be problems in the detecting subtle changes in amino acid levels with in vivo microdialysis technique, because these differences could be due to differences in the efficiency of probe recovery between these groups. Given there are limitations to the microdialysis technique, the present results of measurements of Glu and GABA should be interpreted with caution.

In conclusion, morphine-induced CPP in rats was accompanied by increased Glu and reduced GABA levels in mesocorticolimbic regions, but NAc stimulation led to reversal, with a significant decline in Glu levels and increase in GABA levels, that are likely to reflect inhibition of the mesocorticolimbic reward circuit. These findings provided new insights into the mechanism that underlie the efficacy of deep brain stimulation in the treatment of drug addiction.

## Supporting Information

Figure S1 **Diagram of the experimental procedure.**
(TIF)
