# Supplementary figures and images for: High-Frequency Stimulation of Nucleus Accumbens Changes in Dopaminergic Reward Circuit
Source: PLoS One. 2013 Nov 14;8(11):e79318. doi: 10.1371/journal.pone.0079318 (PMC3828386; doi:10.1371/journal.pone.0079318)

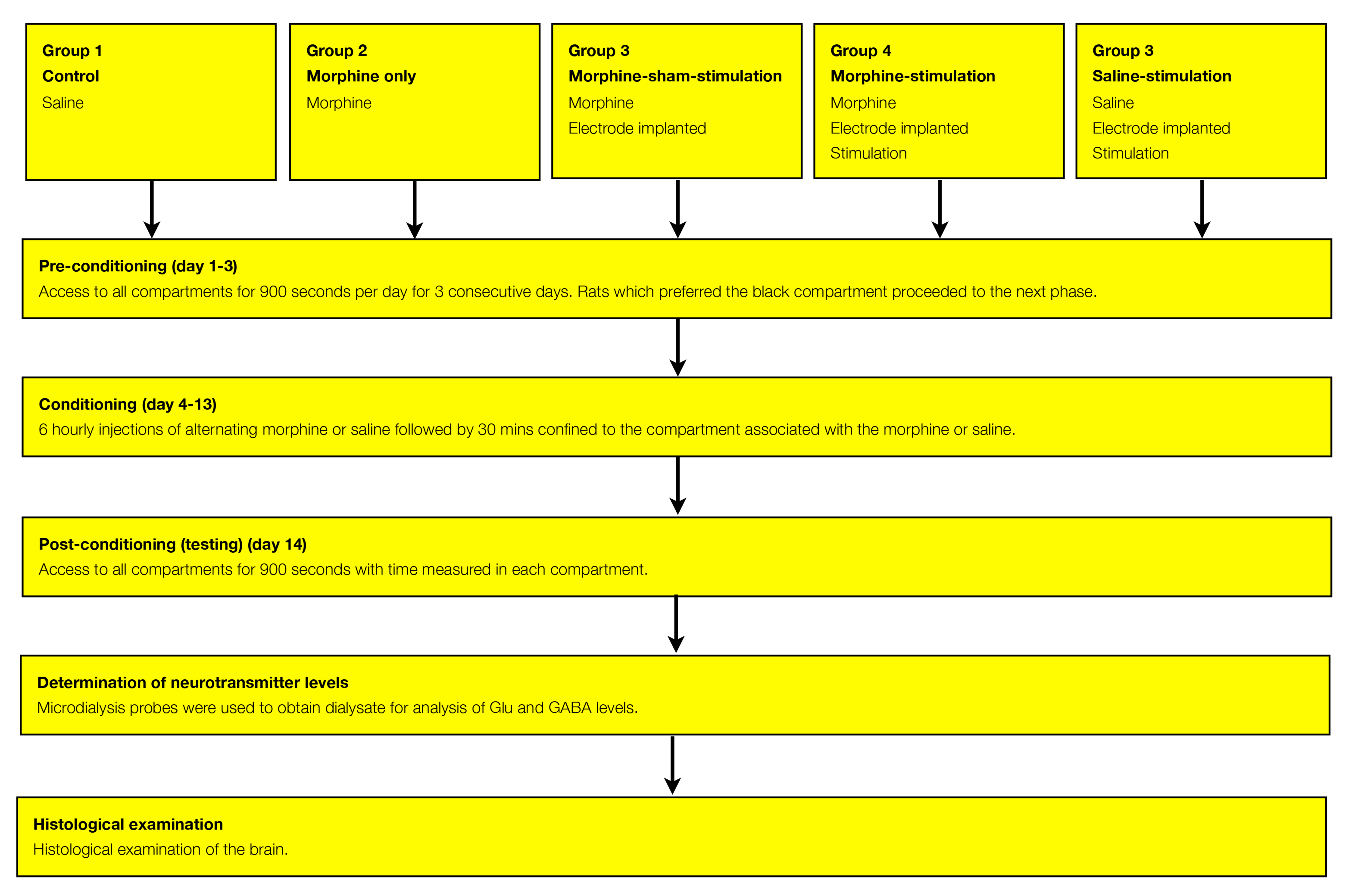

Supplement: Figure S1 — Diagram of the experimental procedure. (TIF) [file pone.0079318.s001.tif]
